# An Analysis of the Biocompatibility, Cytotoxicity, and Bone Conductivity of Polycaprolactone: An In Vivo Study

**DOI:** 10.3390/polym16162271

**Published:** 2024-08-10

**Authors:** Wâneza Dias Borges Hirsch, Alexandre Weber, Janaine Ferri, Adriana Etges, Paulo Inforçatti Neto, Frederico David Alencar de Sena Pereira, Cláiton Heitz

**Affiliations:** 1 Department of Stomatology, School of Dentistry, Federal University of Santa Maria, Santa Maria 97105-340, Brazil; alexandreweber@outlook.com; 2 School of Dentistry, Pontifícia Universidade Católica do Rio Grande do Sul (PUCRS), Porto Alegre 90610-970, Brazil; jsferri@ig.com.br (J.F.); claitonheitz1@gmail.com (C.H.); 3 School of Dentistry, Universidade Federal de Pelotas, Pelotas 96010-610, Brazil; aetges@ufpel.tche.br; 4 Centro de Tecnologia da Informação Renato Archer, Campinas 13069-901, Brazil; paulo.inforcatti@cti.gov.br (P.I.N.); freddasp@gmail.com (F.D.A.d.S.P.)

**Keywords:** bone tissue engineering, biocompatibility, biomaterials, polycaprolactone, animal model

## Abstract

Background: Tissue engineering represents a promising field in regenerative medicine, with bioresorbable polymers such as polycaprolactone (PCL) playing a crucial role as scaffolds. These scaffolds support the growth and repair of tissues by mimicking the extracellular matrix. Objective: This study aimed to assess the in vivo performance of three-dimensional PCL scaffolds by evaluating their effects on bone repair in rat calvaria and the tissue reaction in subcutaneous implant sites, as well as their impact on major organs such as the kidneys, lungs, and liver. Methods: Three-dimensional scaffolds made of PCL were implanted in the subcutaneous tissue of rats’ backs and calvaria. Histological analyses were conducted to observe the bone repair process in calvaria and the tissue response in subcutaneous implant sites. Additionally, the kidneys, lungs, and livers of the animals were examined for any adverse tissue alterations. Results: The histological analysis of the bone repair in calvaria revealed newly formed bone growing towards the center of the defects. In subcutaneous tissues, a thin fibrous capsule with collagenous fibers enveloping the implant was observed in all animals, indicating a positive tissue response. Importantly, no harmful alterations or signs of inflammation, hyperplasia, metaplasia, dysplasia, or hemorrhage were detected in the kidneys, lungs, and liver. Conclusions: The findings demonstrate that PCL scaffolds produced through additive manufacturing are biocompatible, non-cytotoxic, and bioresorbable, promoting osteoconduction without adverse effects on major organs. Hence, PCL is confirmed as a suitable biomaterial for further studies in tissue engineering and regenerative medicine.

## 1. Introduction

Bioresorbable polymers have been used as scaffolds (support) for cell cultures in tissue engineering, thus representing an important alternative for the treatment of lesions and tissue losses [1]. The polymer named polycaprolactone (PCL), a dense and porous type of support, is prepared with specific characteristics that allow for cell growth and proliferation, as well as the formation of new tissue. It is described as a biodegradable and bioresorbable material with very well-established indications [2,3,4], having a melting point between 58 and 63 degrees Celsius (°C) and an elastic modulus of 0.4 gigapascal (GPa). Additionally, its time of degradation ranges from 24 to 36 months [5,6].

Furthermore, biomaterials like PCL have properties that are of great interest for tissue engineering, such as time of degradation, porosity, biocompatibility, and mechanical resistance. Scaffolds from these materials may be made with a variety of shapes and sizes [4,7,8].

The processes of biodegradation and bioresorption have a complex mechanism of cellular and biochemical events. With the implantation of a synthetic material, the organism promotes an inflammatory reaction to the foreign body. The influence of bioresorbable polymers on the degradation due to the presence of peroxides, enzymes, and phagocytic cells represents an important focus of research on bioresorbable polymers [2,9].

This study used PCL to structure three-dimensional scaffolds by means of an experimental platform made on the Fab@CTI additive manufacturing machine, which has an interchangeable extrusion head designed to allow the material to be inserted as a filament. From then on, scaffolds may be prototyped in different shapes and sizes [10].

Bioabsorbable polymers, such as PCL, are alternative materials for the treatment of lesions and tissue losses. They have great potential of use, in addition to presenting mechanical characteristics similar to those of biological materials. These polymers allow for cell growth and proliferation, as well as for the formation of new tissue [3,8,11,12].

In order to contribute to the study on bone substitutes, this paper aimed to observe their biocompatibility by analyzing the reactions between prototyped PCL scaffolds and the subcutaneous tissues of rats’ backs. It also aimed to assess systemic toxicity by analyzing the animals’ livers, lungs, and kidneys 60 days after surgery by microscopic analysis, as well as 7, 21, 60, 90, and 120 days after surgery in animals that received a calvarial implant.

## 2. Materials and Methods

The present study was approved by the Pontifical Catholic University of Rio Grande do Sul (protocol no. 10/00204) where it was conducted, and animal care was in accordance with the institution’s guidelines. Thirteen 120-day-old male Wistar rats weighting between 250 and 300 g were used.

During the entire experiment, all animals were given water and Nuvital^®^ (Nuvital Nutrientes S/A, Curitiba, Brazil) chow ad libitum and were housed in a vivarium in ventilated shelves equipped with input and output air filters (Alesco Ltd., Monte Mor, Brazil), at a controlled temperature (22 + 1 °C) and a dark–bright cycle of 12 h (lights are turned on at 7 a.m. and turned out at 7 p.m.). Rats were kept in standard cages filled with pine wood chips, which were changed three times a week, properly identified according to the group the animals belonged to, and containing at most six animals per cage.

Rats were randomly distributed into two groups, one with five animals (group 1) and another with six animals (group 2). In group 1, systemic toxicity was evaluated by analyzing their organs according to the time when the animals were euthanized: 7, 21, 60, 90, and 120 days after surgery, with PCL being inserted into the bone defect of each animal’s calvarium.

In group 2, biocompatibility and systemic toxicity were assessed 60 days after surgery for PCL scaffold implantation on the rats’ backs by observing the animals’ tissue responses to the implanted biomaterial and by analyzing their organs. PCL implants were subcutaneously inserted into animals’ backs with the preparation of surgical cavities in the subcutaneous connective tissue. The left (experimental) cavity was filled with PCL, while the right (control) cavity did not receive any material, because it acted as a control cavity for wound repair.

In the control group, which included two animals, PCL was not implanted, so their organs were used for the sake of comparison to evaluate tissue alterations in the organs of the animals that received the implants.

After being weighed on a precision scale, the animals were anesthetized by an intraperitoneal injection of a mixture of ketamine hydrochloride (ketamin^®^, Cristália Produtos Químicos Farmacêuticos Ltd., Itapira, Brazil) (100 mg/kg) and xylazine hydrochloride (calmiun^®^, Agener União, São Paulo, Brazil) (10 mg/kg). Once anesthesia was induced, hairs were removed from the upper region of the head located between the external ears in animals of group 1, and from the back in animals of group 2, using an electric hair trimmer (Panasonic^®^ ER389K mustache and beard trimmer, Osaka, Japan). Subsequently, the hairless region and the surrounding coat underwent antisepsis with 0.12% chlorhexidine digluconate. Next, animals received local anesthesia by subcutaneous anesthetic infiltration with 2% lidocaine chlorhydrate and 1:50.000 norepinephrine (Lidostesim 2%, Probem^®^, Catanduva, Brazil) in order to achieve hemostasis and additional analgesia during surgery, besides controlling pain at the immediate postoperative period.

After anesthetic infiltration, animals from group 1 received a coronal linear incision between their two ears, which was made with scalpel blade no. 15 (Solidor, São Paulo, Brazil) mounted on Bard Parker scalpel handle no. 3 (Schobell Industrial Ltd., Rio Claro, Brazil) and measuring around 1.5 cm in size, always supported by a bone base. After this procedure, soft tissues of the head were retracted using two Farabeuf retractors (Schobell Industrial Ltd. Rio Claro, Brazil), providing good visualization of the periosteum, which was incised, divulsed by a Molt retractor and retracted along with the remaining tissues, thus exposing the external surface of the calvarium. Subsequently, the region was irrigated with 0.9% saline using a 20 mL disposable syringe and then dried with sterile gauze.

**Figure 1:**
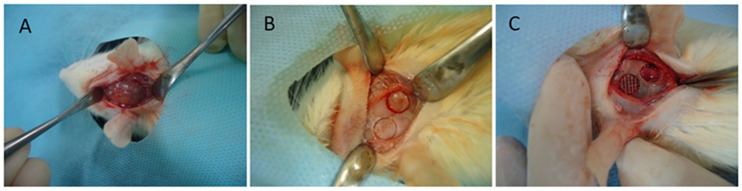
(**A**) Incision in rat’s calvarium. (**B**) Bone defects prepared with bone trephine. (**C**) Experimental bone defect filled with polycaprolactone disc and empty control defect.

Two bone defects were prepared using an electric motor rotating at low speed and bone trephine measuring 5 mm in diameter, which corresponded to the size of the bone defects created during surgery (Figure 1). After being prepared, cavities were abundantly irrigated with saline to remove the residues produced in the process of defect preparation and dried with sterile gauze. PCL was inserted into the cavities located on the left side of calvaria using Adson Brown forceps (Schobell Industrial Ltd., Rio Claro, Brazil). Control cavities were prepared on the right side of calvaria and filled with blood cloth (Figure 1).

**Figure 2:**
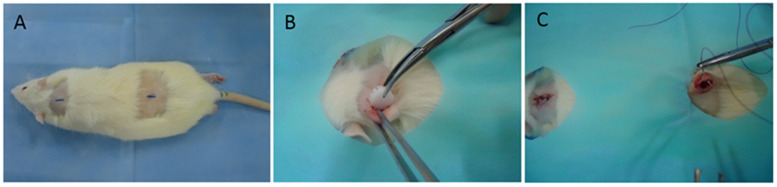
(**A**) Incisions at midline on rat’s back. (**B**) Insertion of a polycaprolactone disc into surgical cavity. (**C**) Suture of dorsal tissues.

After anesthetic infiltration, animals from group 2 received two midline incisions that were equidistant from tail and head insertions and located 7 cm apart from each other. Incisions measured approximately 8 mm in length and were made using scalpel blade no. 15 (Solidor, São Paulo, Brazil) mounted on Bard Parker scalpel handle no. 3 (Schobell Industrial Ltd., Rio Claro, Brazil). The subcutaneous tissue was laterally divulsed with rounded-point scissors in order to form surgical cavities with approximately 18 mm in depth. Subsequently, each PCL implant was inserted into the experimental cavity until reaching its entire depth using Adson Brown forceps (Schobell Industrial Ltd., Rio Claro, Brazil). Special care was taken not to perforate or lacerate the rats’ tissues. Implants were carefully inserted in a non-parallel fashion to the incision line, with the purpose of preventing their expulsion or mobility (Figure 2).

The PCL (CAPA^®^ 6505 polycaprolactone) used in this research, whose chemical formula is (C_6_H_10_O_2_), was synthesized by Solvay Interox Limited, Warrington, UK. According to manufacturer’s recommendations, this material can be used to produce several products, including adhesives, films, fixation agents, and blocks.

Soft tissues were then repositioned so that the periosteum covered bone cavities, and incision edges were sutured with suture thread 5-0 (Johnson & Johnson, Sorocaba, Brazil), performing single interrupted stitches using a Mayo Hegar needle holder and Adson Brown forceps (Schobell Industrial Ltd., Rio Claro, Brazil). Afterwards, the surgical area was cleaned with gauze dampened with saline to remove blood residues, and animals were placed in the prone position in their corresponding cages to recover from anesthesia.

Postoperative pain was controlled with paracetamol (Tylenol^®^ JANSSEN-CILAG Farmacêutica, São Paulo, Brazil) (80 mg/kg) given orally immediately after the procedure and after 12 h. All animals were given a single intramuscular dose of penicillin G benzathine (Benzetacil, Eurofarma Laboratórios Ltd., São Paulo, Brazil) (20,000 units/kg) immediately after the end of the procedure.

After the end of the postoperative observation period proposed for each group, animals were euthanized by isoflurane inhalation. Hairs from the regions of interest were removed using an electric hair trimmer (Panasonic^®^ ER389K mustache and beard trimmer, Osaka, Japan) and then these areas underwent antisepsis with 0.12% chlorhexidine digluconate.

Specimens from animals of group 1 were obtained through an incision in the most posterior region of soft tissues of the head using scalpel blade no. 15 mounted on Bard Parker scalpel handle no. 3 (Schobell Industrial Ltd., Rio Claro, Brazil). The soft tissue overlying the calvarium was removed using Metzenbaum scissors and Adson Brown (Schobell Industrial Ltd., Rio Claro, Brazil), which made it possible to achieve a great visualization of the calvarium, including parietal bones. Subsequently, the calvarium was removed by osteotomy using conical stem multilaminated drill no. 701 rotating at low speed and under constant irrigation with 0.9% saline. Four osteotomy lines were drawn around bone defects and the calvarium was removed using a straight chisel and Adson Brown forceps (Schobell Industrial Ltd., Rio Claro, Brazil). In order to evaluate systemic toxicity, the animals’ livers, lungs, and kidneys were removed through an abdominal incision for histological analysis.

Specimens from animals in group 2 were obtained through excision biopsy of the implant area after the implant was located by palpation. This biopsy was performed with a safety margin of 1 cm and began with an incision using scalpel blade no. 15 mounted on Bard Parker scalpel handle no. 3 (Schobell Industrial Ltd., Rio Claro, Brazil). The dorsal subcutaneous tissue was divulsed using Metzenbaum scissors and Adson Brown forceps (Schobell Industrial Ltd., Rio Claro, Brazil), which made it possible to achieve a great visualization of the calvarium, including the PCL implant and a sufficient amount of normal adjacent tissue. In order to evaluate systemic toxicity, the animals’ livers, lungs, and kidneys were removed for histological analysis. After local macroscopic examination, specimens were immediately stored in identified plastic containers and immersed in 10% neutral buffered formalin for tissue fixation and conservation in order to prevent post-mortem alterations in the tissues.

After the specimens were fixed in formaldehyde for more than 24 h and less than 72 h, another stage of the research started: the preparation and analysis of histological slides. Specimens from group 1 were decalcified in 5% nitric acid solution (10 mL) for approximately 72 h and defects were separated between themselves and divided in half. Specimens from the back, belonging to group 2, and from organs used to evaluate systemic toxicity did not require decalcification. Subsequently, standard procedures for staining with hematoxylin and eosin (HE) were performed, as well as the routine histological processing for the preparation of slides, which included paraffin embedding, the performance of four semi-serial sections of approximately 6 µm thickness in each block with a distance of 15 µm between each section, measured on a microtome (Jung RM 2055 microtome, Leica Biosystems, Wetzlar, Germany), HE staining, and examination of the slides on a light optical microscope (BX 50 microscope, Olympus, Melville, NY, USA). Slides were codified in such a way that the observer was unaware of which group they belonged to.

Evaluation was performed by the same previously calibrated examiner. Histological analyses were carried out using a light microscope at 40, 100, and 400× magnifications, distributed into fields scanning all the areas containing PCL.

The analysis and description of the slides were based on the criteria established next. Calvaria containing PCL were assessed for new bone formation originating from the margins of the bone defect or from the center of the bone defect, or located on the edges of the biomaterial, as well as for the presence or absence of material resorption.

Backs containing PCL were microscopically evaluated for cellular and tissue reactions, the presence of fibrous capsules adjacent to the material that had been implanted and its thickness, the presence of inflammatory infiltrate and of inflammatory multinucleated giant cells, vascular alterations, and the formation of granulation tissue. The fibrous capsule was defined as thin or thick; the granulation tissue as young or mature; fibrosis as organized or disorganized; and finally, vasodilatation, hyperemia, and edema were defined as mild, moderate, or severe. Moreover, the inflammatory infiltrate located close to the material under analysis was defined as absent when the percentage of inflammatory cells was up to 10%; moderately present if the presence of inflammatory cells was observed, but they did not dominate the histological field in analysis, with a percentage ranging from 10 to 50%; and severely present when the cells form an infiltrate around the bone portion to be observed, with a percentage higher than 50% [13].

According to Souza et al. [14], experimental materials are considered biocompatible if the intensity of the inflammatory reaction in the connective tissue decreases over time. Therefore, after microscopic evaluation of specimens for 60 days, the material under investigation was considered biocompatible when the sample has a thin layer of fibrous capsule around the implant and there was no evidence of an inflammatory reaction, macrophages or inflammatory multinucleated giant cells. On the other hand, it was considered non-biocompatible when there was a persistent inflammatory reaction related to macrophages and giant cells, as well as the development of a thick fibrous capsule.

Additionally, each animal was assessed for systemic toxicity by investigating liver, kidney, and lung changes, such as the presence of cellular or inflammatory infiltration and tissue alterations like hyperplasia, metaplasia, and/or dysplasia. No statistical tests were applied, since it was a qualitative study.

## 3. Results

**Figure 3:**
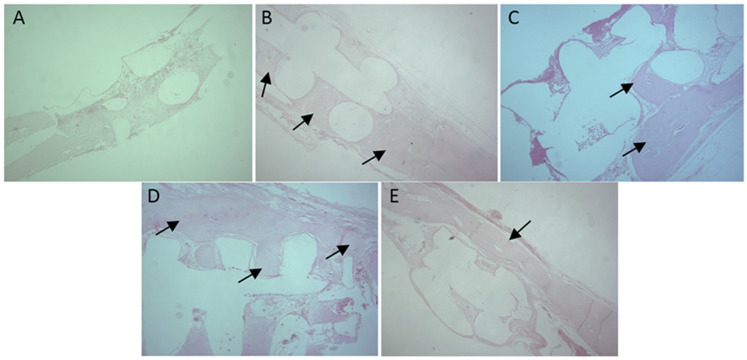
Histologic images of new formed bone in defects containing biomaterial at 7 days (**A**), 21 days (**B**), 60 days (**C**), 90 days (**D**), and 120 days, showing the formation of a bone bridge (**E**). Areas of new bone formation (arrows).

In group 1, specimens from calvaria containing PCL implants were investigated through histological analysis, and systemic toxicity was observed through the analysis of animals’ organs. It was found that there was new bone formation after 21 days of postoperative follow-up, which means that the area of newly formed bone gradually increased over 60, 90, and 120 days (Figure 3). In all animals, new bone formation originated from the margins of the bone defect. New bone formation in the borders of the biomaterial and PCL resorption were also observed.

An analysis of the events occurring in the kidneys, livers, and lungs from the animals of group 1 showed that there were no tissue alterations that could damage these organs.

No presence of inflammatory processes, hyperplasia, metaplasia, dysplasia or hemorrhage was observed in rats’ kidneys. There were no cases of tubular necrosis. The only alterations found in these animals were mild glomerular hypercellularity, vascular congestions, and foci of capillary aggregates, which also appeared in control animals.

There were no signs of inflammatory processes, hyperplasia, metaplasia, dysplasia or hemorrhage in animals’ livers as well. In addition, no microvesicular steatosis, necrosis or apoptosis were observed. There were only very few cells with macrovesicular steatosis or vascular and sinusoidal congestions, events that were also observed in control animals.

**Figure 4:**
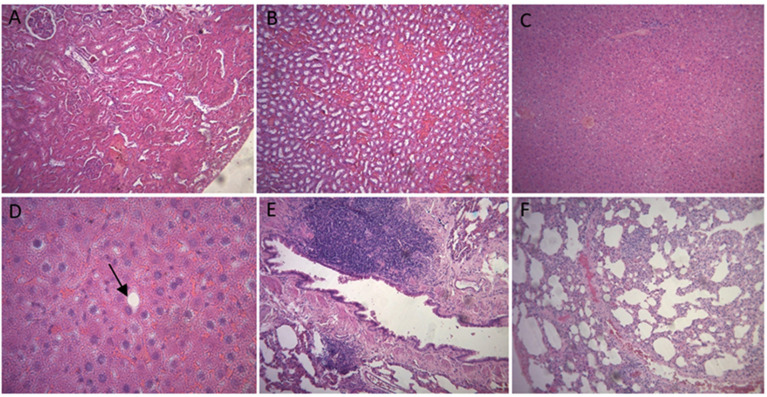
Histologic images of animals’ organs. Kidney with mild glomerular hypercellularity (**A**), kidney with vascular congestion and foci of capillary aggregates (**B**), liver with vascular and sinusoidal congestion (**C**), liver with cells presenting with macrovesicular steatosis (arrow) (**D**), lung with peribronchial lymphoid aggregates (**E**), and lung with mild alveolar septal thickening and vascular congestion (**F**).

No presence of inflammatory processes, hyperplasia, metaplasia, dysplasia or hemorrhage was found in animals’ lungs. The only significant finding was the presence of peribronchial lymphoid aggregates, alveolar septal thickening, and vascular congestion, events that were also observed in control animals (Figure 4).

In the animals of Group 2, specimens from the backs of rats containing a PCL implant were examined to assess biocompatibility and conduct organ analysis for toxicity assessment.

**Figure 5:**
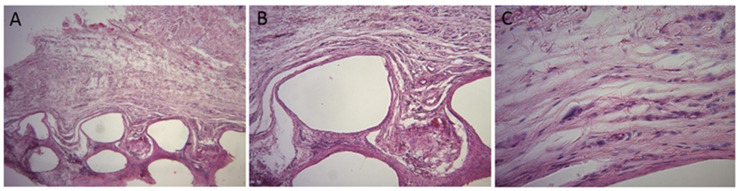
Histologic images of tissues adjacent to the disc implanted on animals’ backs at 60 days. Formation of a thin fibrous capsule involving the implant (**A**), detail of the fibrous capsule, with organized collagen fibers involving the implant (**B**,**C**).

When tissues adjacent to the disc implanted on animals’ backs were observed after 60 days, the formation of a thin fibrous capsule was found in all animals, with organized collagenous fibers involving the implant (Figure 5). There were no signs of inflammatory infiltrate, granulation tissue, vasodilation, hyperemia, edema or abscess 60 days after the discs were implanted.

**Figure 6:**
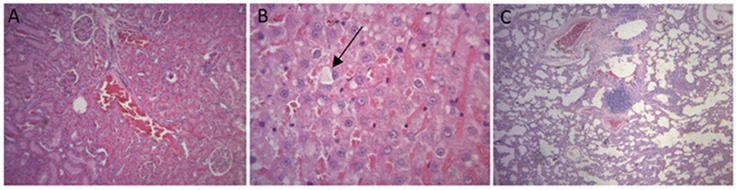
Histologic images of animals’ organs. Kidney with mild glomerular hypercellularity and vascular congestion (**A**), liver with vascular and sinusoidal congestion and cell presenting with macrovesicular steatosis (arrow) (**B**), and lung with peribronchial lymphoid agglomerates, mild alveolar septal thickening, and vascular congestion (**C**).

When it comes to events occurring in the kidneys, lungs, or livers of the animals from group 2, no harmful tissue alterations were reported. No inflammatory processes, hyperplasia, metaplasia, dysplasia or hemorrhage were observed in animals’ kidneys, lungs, and livers. Their kidneys did not present with tubular necrosis, and only cases of mild glomerular hypercellularity, vascular congestion, and foci of capillary aggregates were found. Their livers did not develop microvesicular steatosis, necrosis or apoptosis. There were only a few isolated cells with macrovesicular steatosis and vascular and sinusoidal congestion. Rats’ lungs showed peribronchial lymphoid aggregates, mild punctual alveolar septal thickening, and vascular congestion, events that were also observed in the two control animals (Figure 6).

## 4. Discussion

The use of materials to improve or repair the body dates back to antiquity, when natural materials such as wood were used in an attempt to structurally replace tissues lost to trauma or disease [15]. Since the 20th century, these natural materials began to be replaced with polymers, which provided better performance, functionality, and reproducibility [15].

Currently, biomaterials are an increasingly important alternative source in bone regeneration. They should ideally be biocompatible and biodegradable, as well as having the appropriate porosity that allows for vascularization and ensures mechanical resistance. Additionally, its degradation products should be non-toxic [16,17,18].

PCL is a type of bioabsorbable polymer that has a great potential for use in bone repair, because it presents mechanical characteristics similar to that of biologic materials, allowing for cell growth and proliferation, as well as the formation of new tissue [3,8,11,12].

The preparation of an appropriate three-dimensional scaffold is essential to determine whether the material can be used as a bone substitute. An ideal scaffold should have pores able to provide enough space for a uniform cell distribution and an appropriate oxygen and nutrient reception, in addition to having good biocompatibility and osteoconductivity [19,20].

In the present study, PCL scaffolds were prototyped in an experimental platform of the Fab@CTI additive manufacturing machine, in order for the material to be initially transformed into filaments to be used on the machine. Therefore, it became necessary to observe the in vivo characteristics of PCL after this whole process [10]. This study evaluated PCL biocompatibility through the histological analysis of tissue reaction to PCL scaffolds implanted on rats’ backs and calvaria, as well as their systemic toxicity through the analysis of the animals’ kidneys, lungs, and livers.

The main advantages of producing scaffolds by additive manufacturing are precision in material deposition and process reproducibility, making it possible to obtain three-dimensional complex structures and to control internal morphology. Additionally, this process takes a short time and has a relatively low cost [10,21].

The methodology used in this study also allowed evaluating tissue reactions in animal models, which is an essential stage to complete the evaluation of this type of material. In areas where PCL scaffolds were implanted, this material became directly in contact with tissue, including bone tissue, similar to what would occur if the biomaterial was clinically applied [22].

Our histological analysis made it possible to assess the presence of newly formed bone on calvaria, showing that new bone formation occurred towards the center of the defects, as well as to qualitatively assess the presence of the remaining portions of the PCL disc [19,23,24]. The results obtained from this analysis showed that new bone formation occurred after 21 days post-implantation, with the formation of a bone bridge from one margin of the defect to another (Figure 3E) but not the total replacement of the biomaterial with bone tissue [25]. Thus, this evaluation made it possible to investigate the beginning of the osteoconduction process, as well as the slow biomaterial resorption and the replacement of PCL with bone [24]. The histological analysis of tissues from animals’ backs at 60 days allowed observing the formation of a thin fibrous capsule in all animals, with organized collagenous fibers involving the PCL implant, which confirmed findings from other studies [26,27].

With regard to the events occurring in animals’ organs, histological analysis did not reveal tissue alterations that could damage their organs, since no signs of inflammatory processes, hyperplasia, metaplasia, dysplasia or hemorrhage were observed in rats’ kidneys, lungs, and livers.

Some punctual isolated alterations were found, such as mild glomerular hypercellularity and vascular congestion in the kidneys; isolated cells with macrovesicular steatosis and vascular and sinusoidal congestion in the liver; and mild alveolar septal thickening and vascular congestion in the lungs. However, these events were also observed in control animals, which did not receive any type of treatment.

Thus, the characteristics observed in the PCL used in the present study corroborate those conceptually necessary for the material to be appropriate for use in tissue repair, since it did not produce an exacerbated inflammatory reaction, was not rejected by the body, and allowed for osteoconduction [19,28,29].

Therefore, in view of the results obtained, it is possible to conclude that PCL scaffolds produced on the Fab@CTI additive manufacturing machine are biocompatible, non-cytotoxic, and bioresorbable products that promote osteoconduction. Hence, PCL seems to be an appropriate biomaterial to be used in other studies aiming to elucidate issues related to this topic and in future clinical trials.

Displayed equations can be inserted where desired making sure they are assigned Word Style “Normal”. Displayed equations can only be one column wide. If the artwork needs to be two columns wide, it must be relabeled as a figure, chart, or scheme and mentioned as such in the text.

## Data Availability

The original contributions presented in the study are included in the article, further inquiries can be directed to the corresponding author.

## Abbreviations

GPa	giga pascal
PCL	polycaprolactone
